# Supplementary material for: Genomic and functional analysis of rmp locus variants in Klebsiella pneumoniae
Source: Genome Med. 2025 Apr 9;17:36. doi: 10.1186/s13073-025-01461-5 (PMC11984045; doi:10.1186/s13073-025-01461-5)
Supplement: Supplementary file 2 — Additional file 2: Table S2 Primers used in this study. Table S3 Distribution of rmp loci by species. Fig. S1 Phylogenetic relationships of the A. rmpA, B. rmpD and C. rmpC genes. Fig. S2 Distribution of rmpADC allelic variants and KpVP-1 associated virulence loci rmpA2, iuc and iro in Klebsiella pneumoniae clonal group 23. Fig. S3 Distribution of rmpADC allelic variants and KpVP-1 associated virulence loci rmpA2, iuc and iro in Klebsiella pneumoniae clonal group 65. Fig. S4 Distribution of rmpADC allelic variants and KpVP-1 associated virulence loci rmpA2, iuc and iro in Klebsiella pneumoniae clonal group 86 [file 13073_2025_1461_MOESM2_ESM.doc]

**Additional File 2.**

**Table S2: Primers used in this work**

**Name Sequence^a^(5’→3’) Use^b^**

KW531 GGCCCCCCCTCGAGGTCGACGATGAATATTGATGGATCAAAG F pKW215, pKW222

KW532 GCTCTAGAACTAGTGGATCCGGAAACAAAAAGCTATACCATC R pKW215

KW533 GGCCCCCCCTCGAGGTCGACGATGAATATTGATGGAGCAAAG F pKW216

KW534 GCTCTAGAACTAGTGGATCCGGAAACCAAAAGTTATACCATC R pKW216, pKW222, pLPT059

KW543 CGGGCCCCCCCTCGAGGTCGACGATGAATATTGATGGTTCAAAG F pLPT059

_____________________________________________________________________________________________

^a^ Blue nt are overhangs for Gibson cloning. Underlined nt indicate restriction sites.

^b^ F, forward primer; R, reverse primer

**Table S3: Distribution of *rmp* loci by species** (number of genomes with typeable *rmp* loci indicated in brackets)

|  | ***K. pneumoniae*** | ***K. quasipneumoniae* subsp. *similipneumoniae*** | ***K. variicola* subsp. *variicola*** |
| --- | --- | --- | --- |
| Number of genomes screened | 11967 | 522 | 626 |
| ***Rmp* status** | | | |
| Any *rmp* sequence detected | 980 (817) | 5 (4) | 7 (4) |
| 1. Functional* *rmp* | 709 (709) | 3 (3) | 4 (4) |
| 1. Incomplete *rmp* | 115 (-) | 1 (-) | 3 (-) |
| 1. Truncated *rmp* | 141 (93) | 1 (1) | - |
| 1. Multiple *rmp* | 15 (15) | - | - |

*Presumably functional; *rmp* loci are complete and intact (i.e. do not carry nonsense mutations)

**Fig. S1: Phylogenetic relationships of the A. *rmpA,* B. *rmpD* and C. *rmpC* genes.** Each tree is a maximum-likelihood phylogeny with each node corresponding to an allelic variant, and includes all allelic variants of *rmpA, rmpD* and *rmpC* that had been defined in the BIGSdb-*Kp* database. Nodes are coloured by the associated mobile genetic element according to the legend. Those marked as “unknown” are allelic variants present in the BIGSdb-*Kp* database but do not match to any genomes and the genetic context is unknown.

Columns are as follows: presence or absence of a truncation, detection within a hypervirulent (blue) or MDR (red) clone or non-*K. pneumoniae* species. The number of genomes from which each allelic variant was detected is shown in the bar graph on the right-hand side.

**
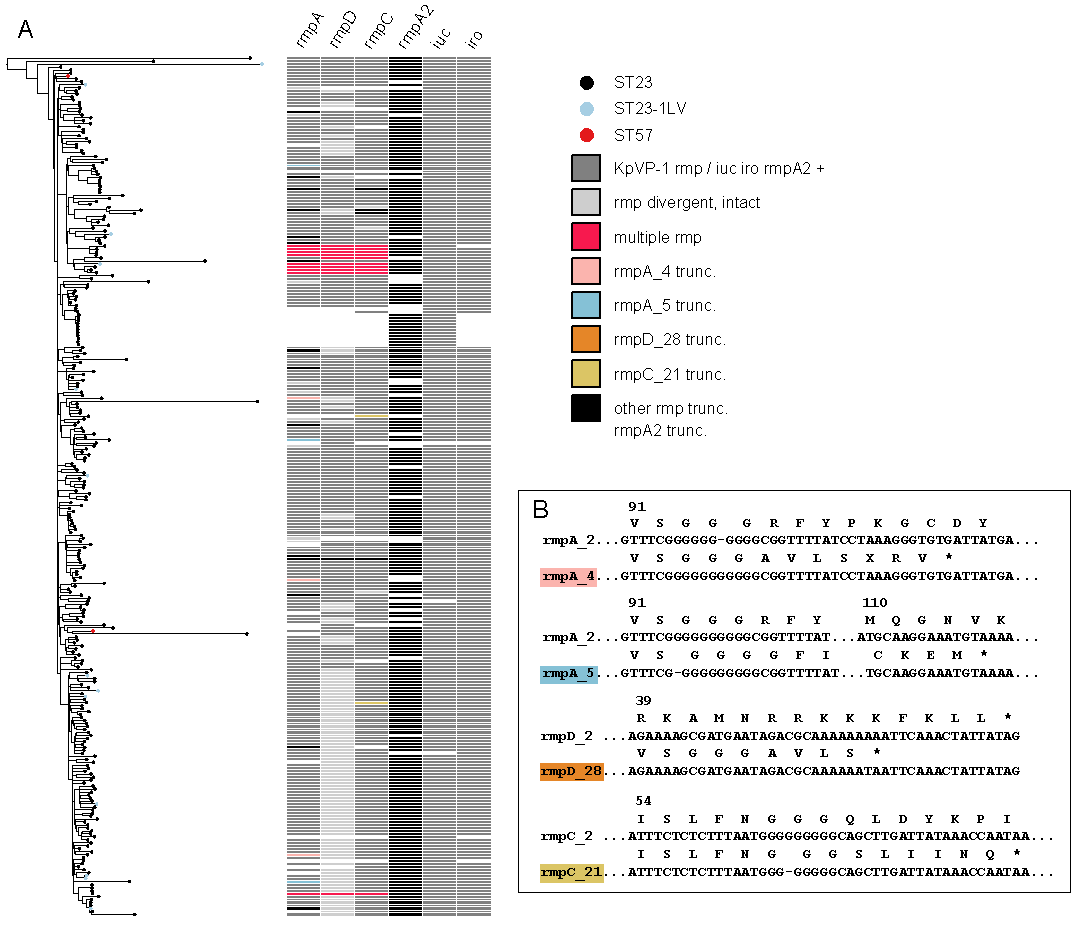
**

**Fig. S2: Distribution of *rmpADC* allelic variants and KpVP-1 associated virulence loci *rmpA2*, *iuc* and *iro* in *Klebsiella pneumoniae* clonal group 23. A.** Tree is a core genome SNP-based neighbour joining tree for 288 CG23 genomes generated from Pathogenwatch. Nodes are coloured by ST as per legend. Column shows the presence or absence (coloured white) of *rmpA, rmpD, rmpC, rmpA2, iuc* and *iro*, and allelic variant or truncation status as specified (coloured according to inset legend). **B**. Nucleotide and amino acid alignments are shown for four allelic variants with indels (i.e. *rmpA_4, rmpA_5, rmpD_28* and *rmpC_21*) compared to the wild-type intact variant (i.e. *rmpA_2*, *rmpD_2* and *rmpC_2*). Asterisk denotes premature stop codon.

**
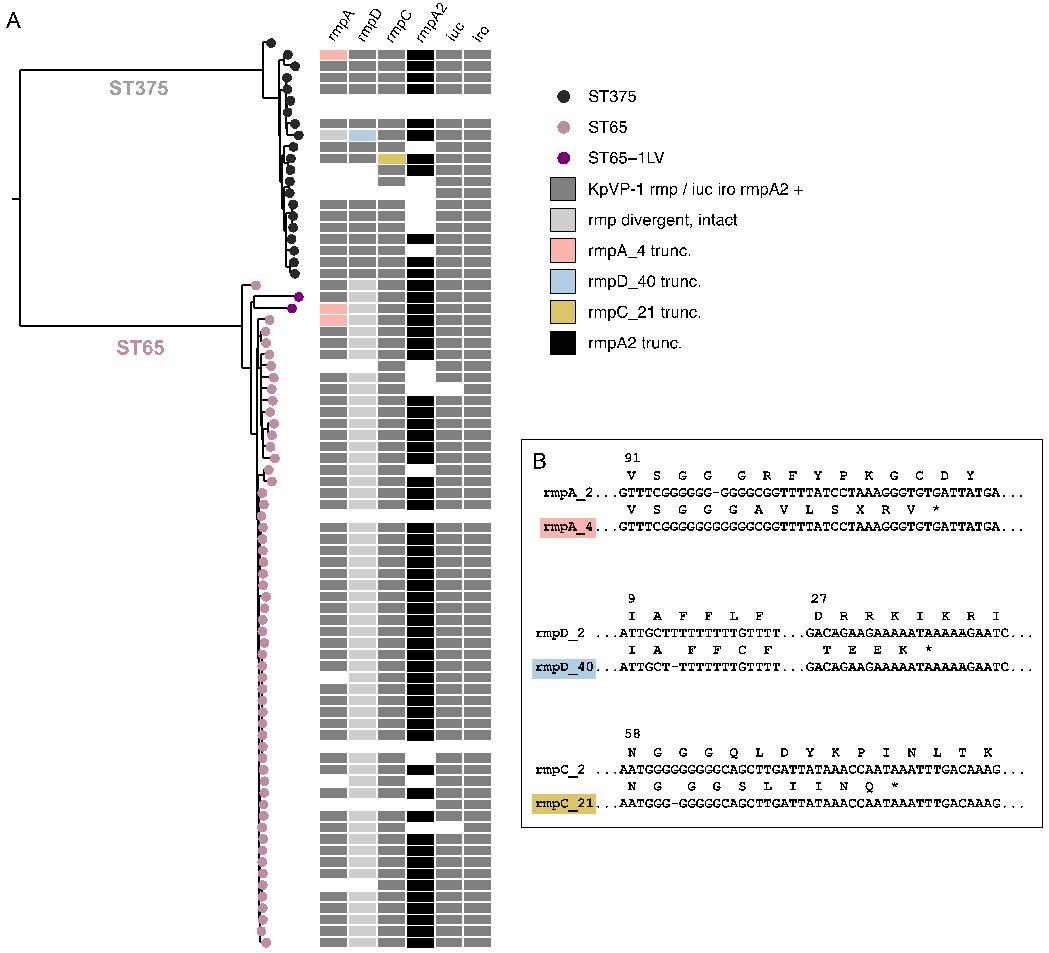
**

**Fig. S3: Distribution of *rmpADC* allelic variants and KpVP-1 associated virulence loci *rmpA2*, *iuc* and *iro* in *Klebsiella pneumoniae* clonal group 65. A.** Tree is a core genome SNP-based neighbour joining tree for 79 CG65 genomes generated from Pathogenwatch. Nodes are coloured by ST as per legend. Column shows the presence or absence (coloured white) of *rmpA, rmpD, rmpC, rmpA2, iuc* and *iro*, and allelic variant or truncation status as specified (coloured according to inset legend). **B.** Nucleotide and amino acid alignments are shown for three allelic variants with indels (i.e. *rmpA_4, rmpD_40* and *rmpC_21*) compared to the wild-type intact variant (i.e. *rmpA_2*, *rmpD_2* and *rmpC_2*). Asterisk denotes premature stop codon.

**
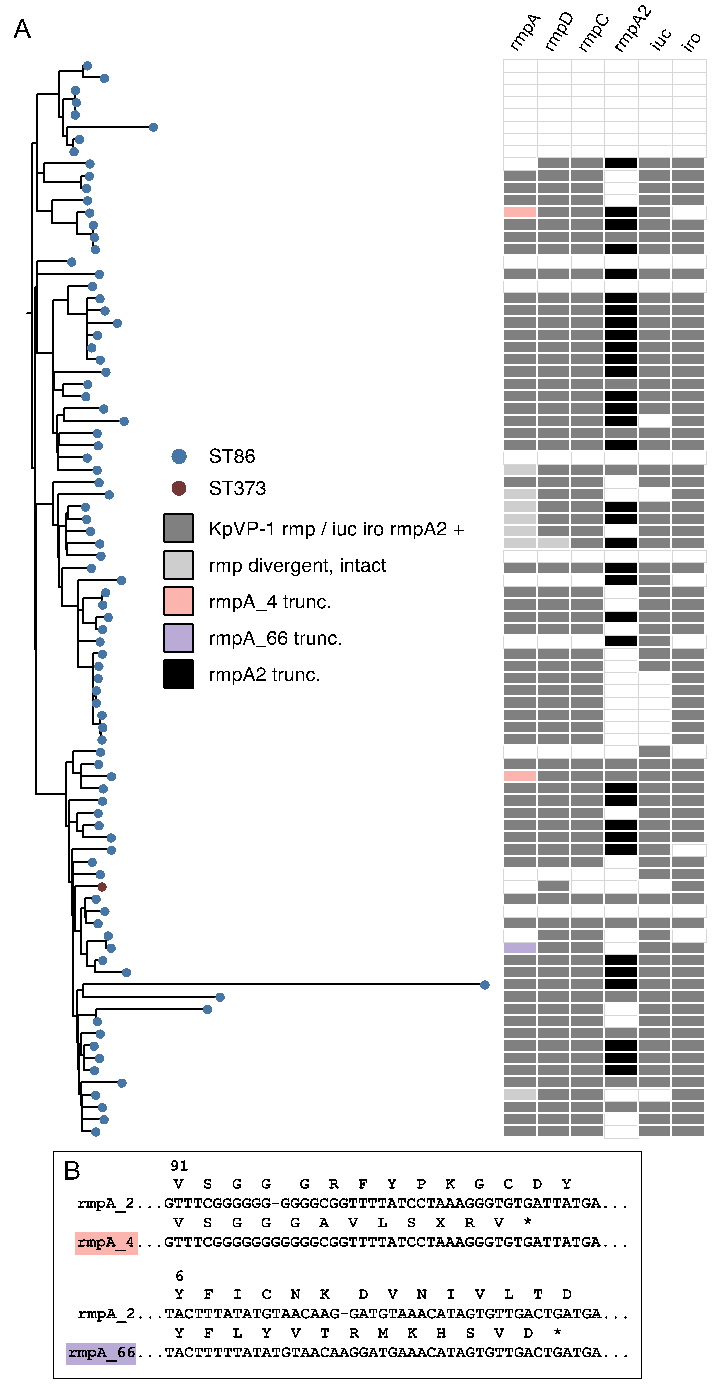
**

**Fig. S4: Distribution of *rmpADC* allelic variants and KpVP-1 associated virulence loci *rmpA2*, *iuc* and *iro* in *Klebsiella pneumoniae* clonal group 86. A.** Tree is a core genome SNP-based neighbour joining tree for 88 CG86 genomes generated from Pathogenwatch. Nodes are coloured by ST as per legend. Column shows the presence or absence (coloured white) of *rmpA, rmpD, rmpC, rmpA2, iuc* and *iro*, and allelic variant or truncation status as specified (coloured according to inset legend). Note that for the subclade comprising eight genomes located towards the top of the phylogeny that lack the virulence plasmid loci of interest, six appear to have lost the plasmid entirely (0-0.5% coverage of pSGH10, accession CP025081.1) while the other two carry variants of the plasmid that lack these loci (95% coverage of pSGH10). **B.** Nucleotide and amino acid alignments are shown for two allelic variants with indels (i.e. *rmpA_4* and *rmpA_66*) compared to the wild-type intact variant (i.e. *rmpA_2*). Asterisk denotes premature stop codon.
